# Supplementary material for: Four-dimensional surface motions of the Slumgullion landslide and quantification of hydrometeorological forcing
Source: Nat Commun. 2020 Jun 3;11:2792. doi: 10.1038/s41467-020-16617-7 (PMC7270131; doi:10.1038/s41467-020-16617-7)
Supplement: Supplementary file 1 — Supplementary Information [file 41467_2020_16617_MOESM1_ESM.pdf]

Supplementary Information for

Four-dimensional surface motions of the Slumgullion landslide and quantification of hydrometeorological forcing

Hu et al.

This file includes:

Supplementary Figures 1-17

Supplementary Tables 1-3

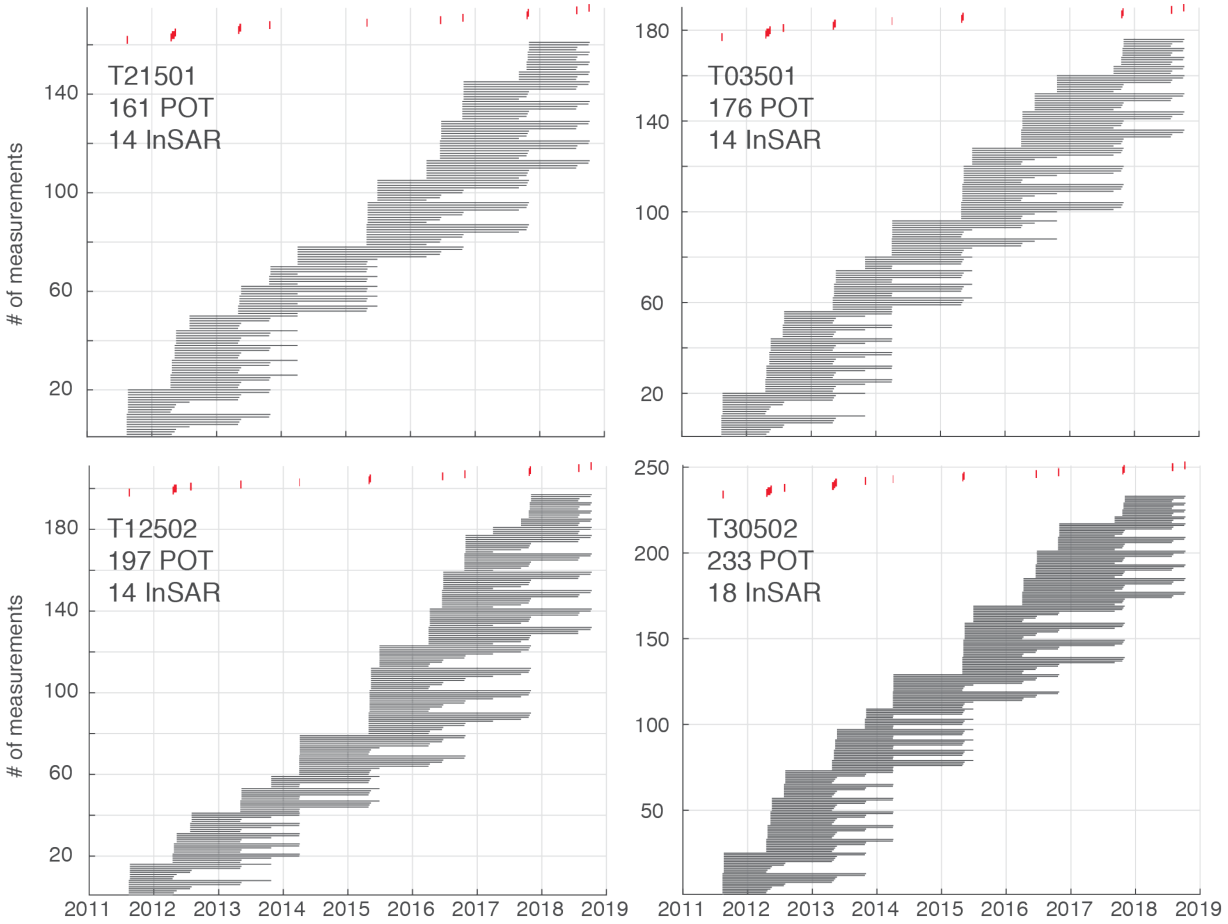

Supplementary Figure 1 | UAVSAR image pair configurations. The pixel offset tracking (POT) method is applied for image pairs shown by black horizontal lines. InSAR processing is applied for the short time intervals shown by red lines at the top of each panel.

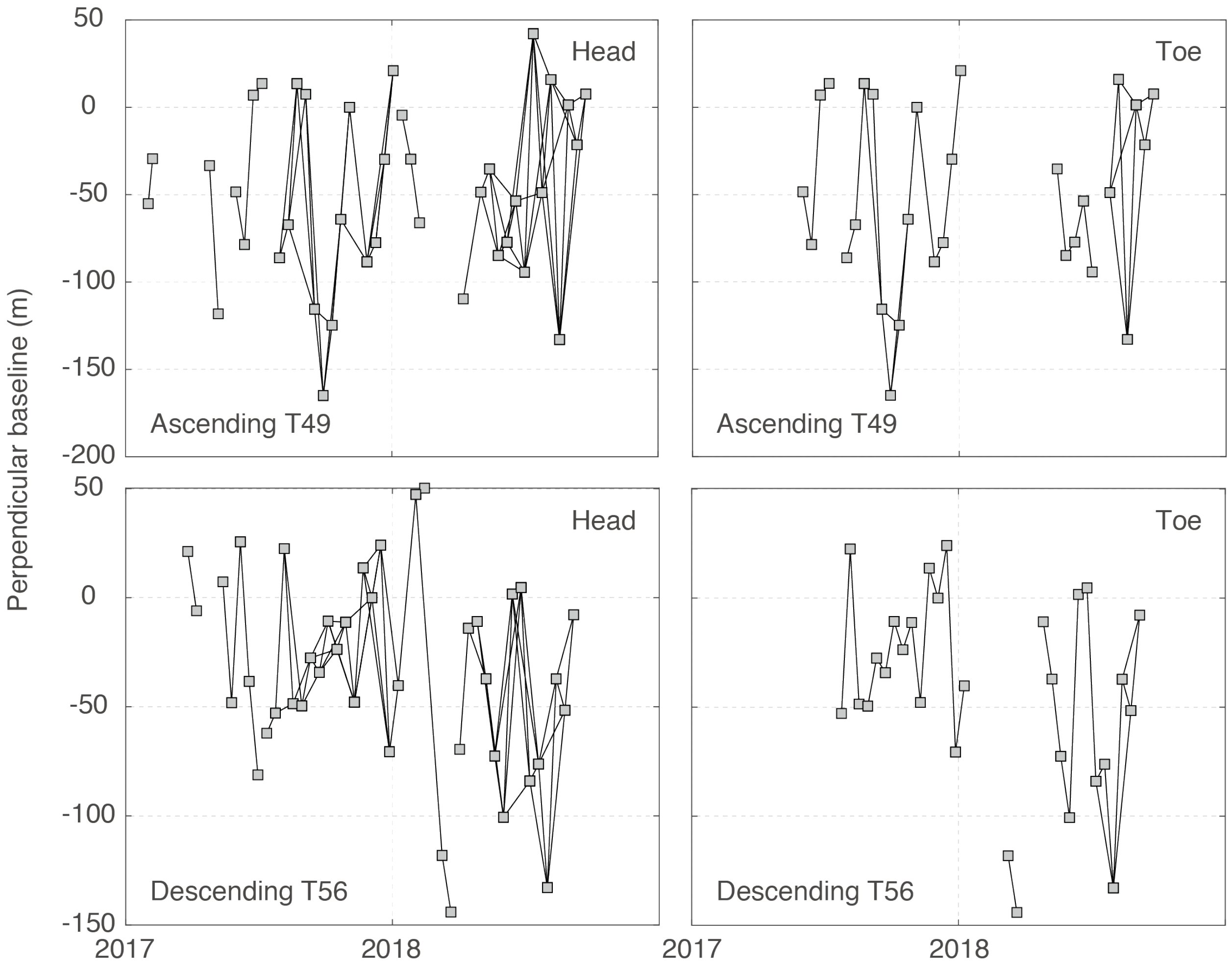

Supplementary Figure 2 | Sentinel-1 InSAR baselines. Symbols connected by lines represent image pairs used to produce interferograms.

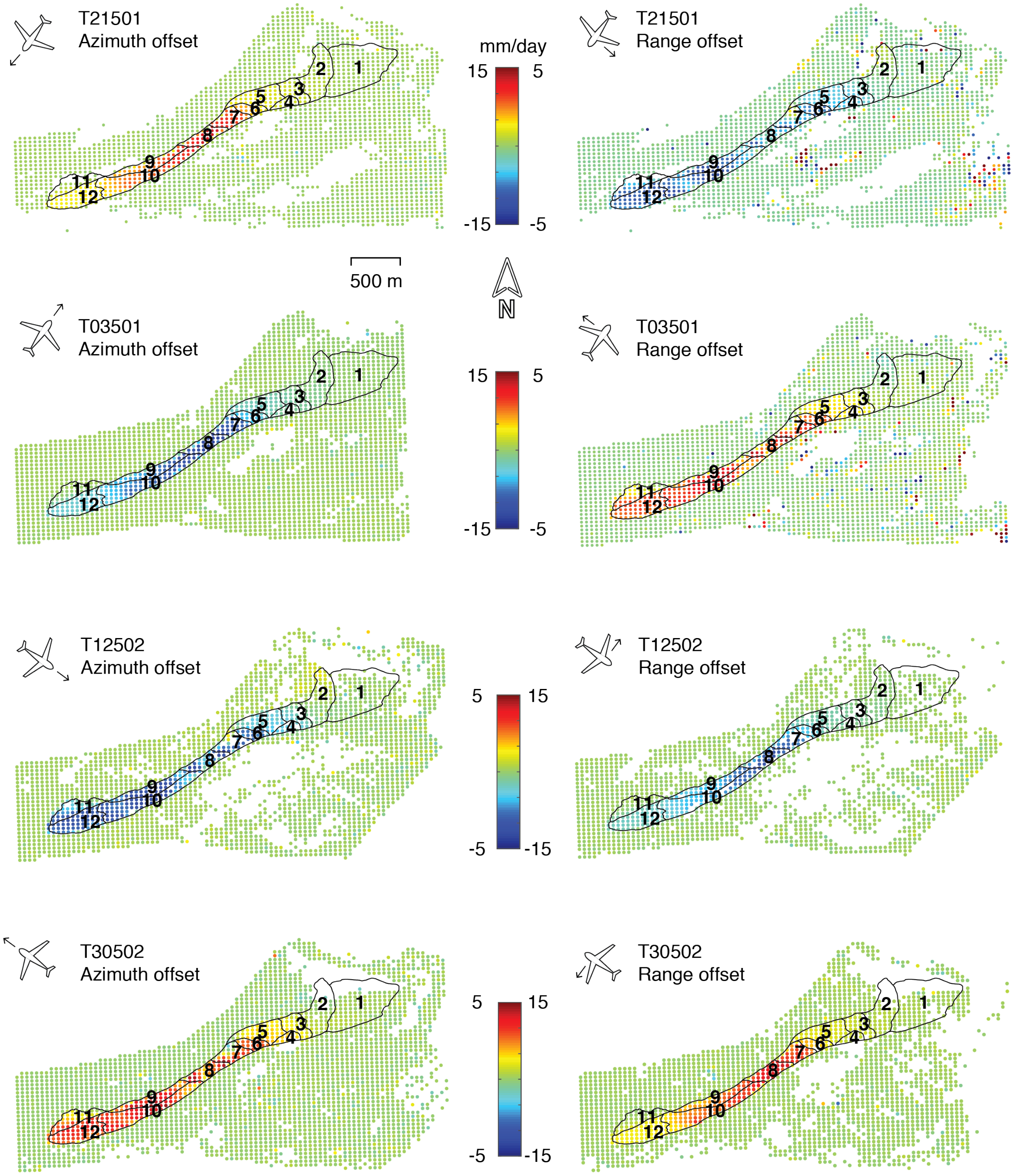

Supplementary Figure 3 | Landslide velocity measured along the specified orientations.

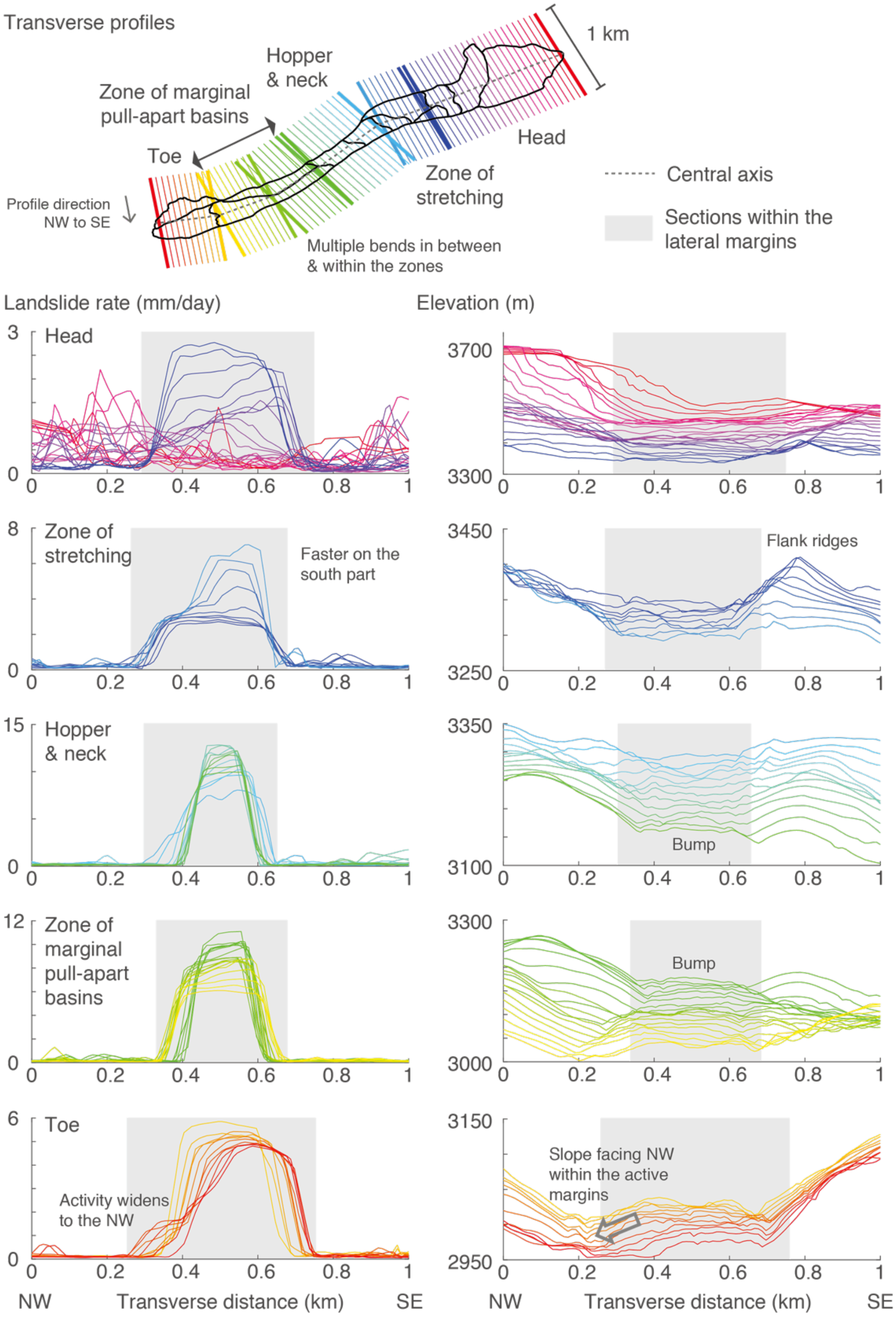

Supplementary Figure 4 | Transverse profiles of longitudinal landslide velocity and surface elevation. The dashed line in the map delineates the central axis. Colored lines show the locations of the 1-km-long transverse profiles. The left column shows longitudinal velocities along each profile and their surface elevation is shown in the right column. Profiles go from the northwest (0 km) to the southeast (1 km). Gray shades indicate the active widths.

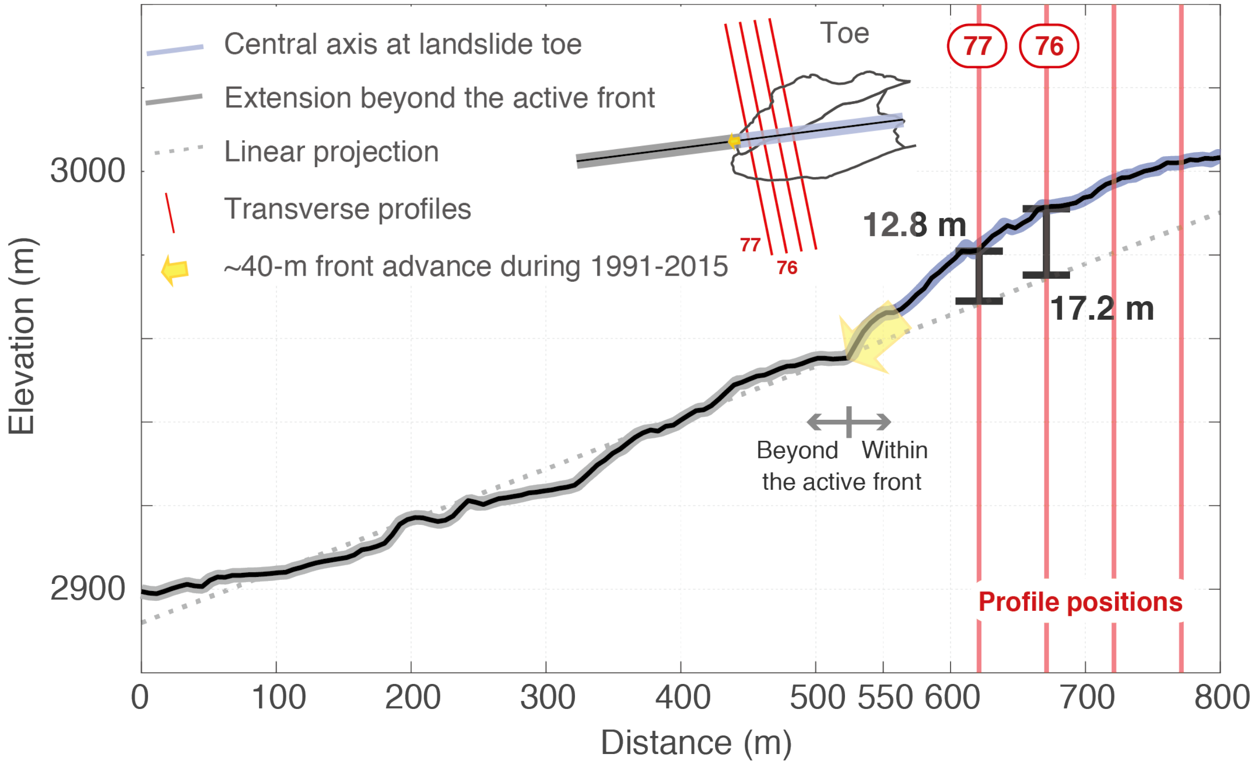

Supplementary Figure 5 | Schematic view of topographic back-projection of the landslide base around the distal toe. Purple lines show the position and the elevation of the neutral axis of the active toe, and gray lines show its extension downhill for 1 km beyond the active landslide front. The elevation data are from the 2015 LiDAR DEM[37]. Dotted line shows the linear fit of elevation of the inactive downhill part and its projection uphill to the active toe. Red lines show the positions of the transverse profiles (see Supplementary Fig. 4). The yellow arrow shows the front advance from the mapped end in 1991[8] to the inferred end referring to the DEM obtained in 2015.

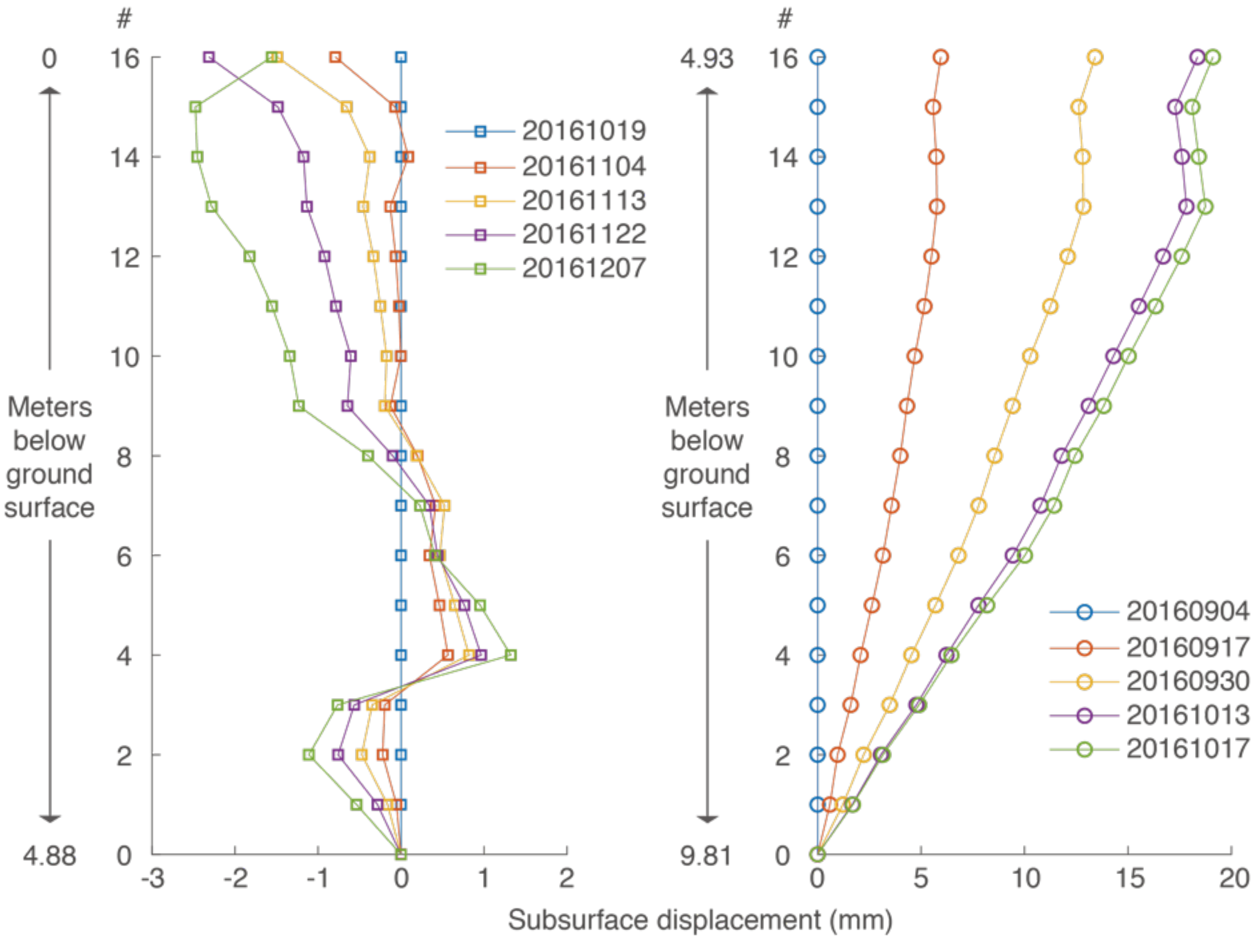

Supplementary Figure 6 | Inclinometer profiles show the subsurface displacement gradients measured at depth 0-4.88 and 4.93-9.81 m by 16 sensors. The measurements are referred to the bottom sensor. Deformation in the upper 4.88 m was less than 2 mm in 49 days. The subsurface drill hole sheared by ~20 mm in 43 days in the deeper profile while the ground surface near the hole moved on average by ~0.4 m monthly with respect to the surrounding terrain.

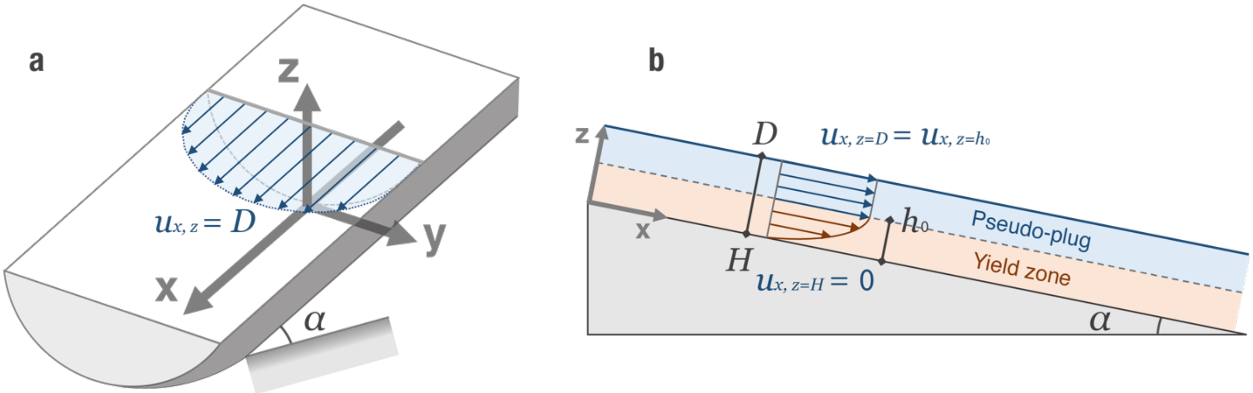

Supplementary Figure 7 | Schematic view of the viscoplastic flow on an inclined plane. (**a**) 3D view and (**b**) side view. x points downhill, y denotes horizontal distance along the cross section from the center of the slide, and z is oriented perpendicular to the inclined basal plane with slope angle α.

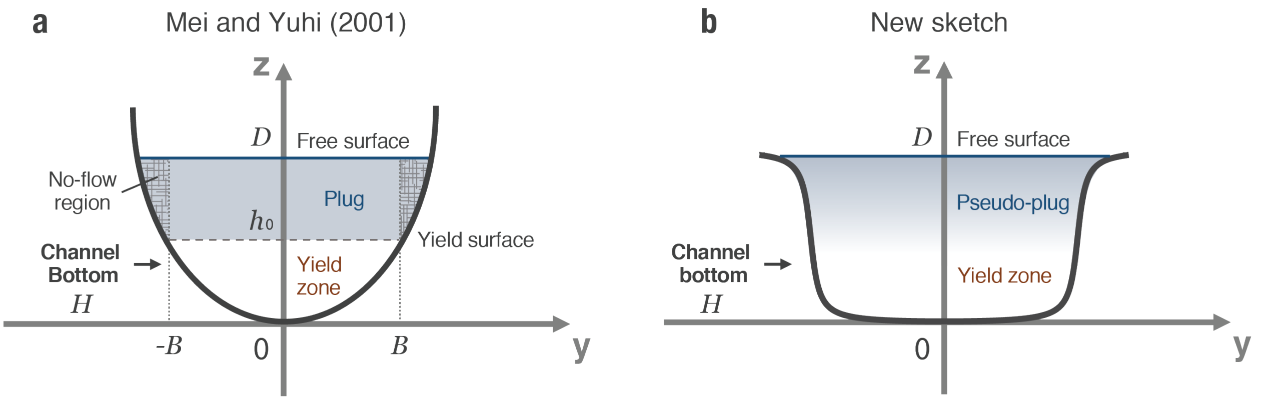

Supplementary Figure 8 | Schematic view of the channel geometry.

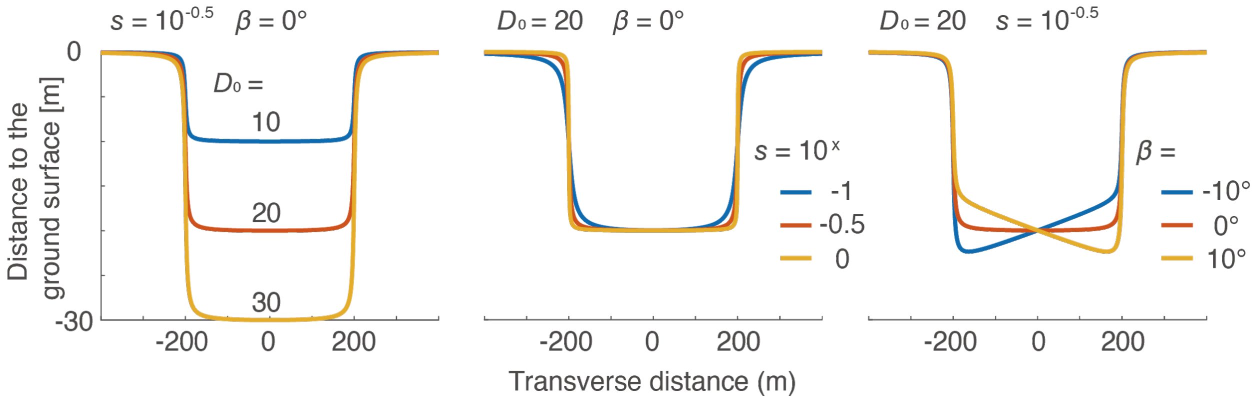

Supplementary Figure 9 | Channel geometry characterized by the depth (D_0), steepness (s), and the basal bed tilt (β).

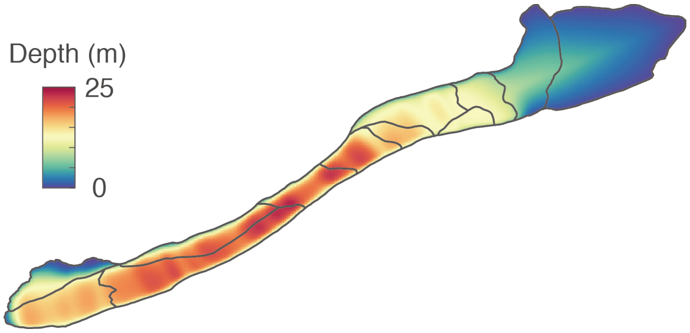

Supplementary Figure 10 | The inferred landslide depth from the power-law viscoplastic flow model.

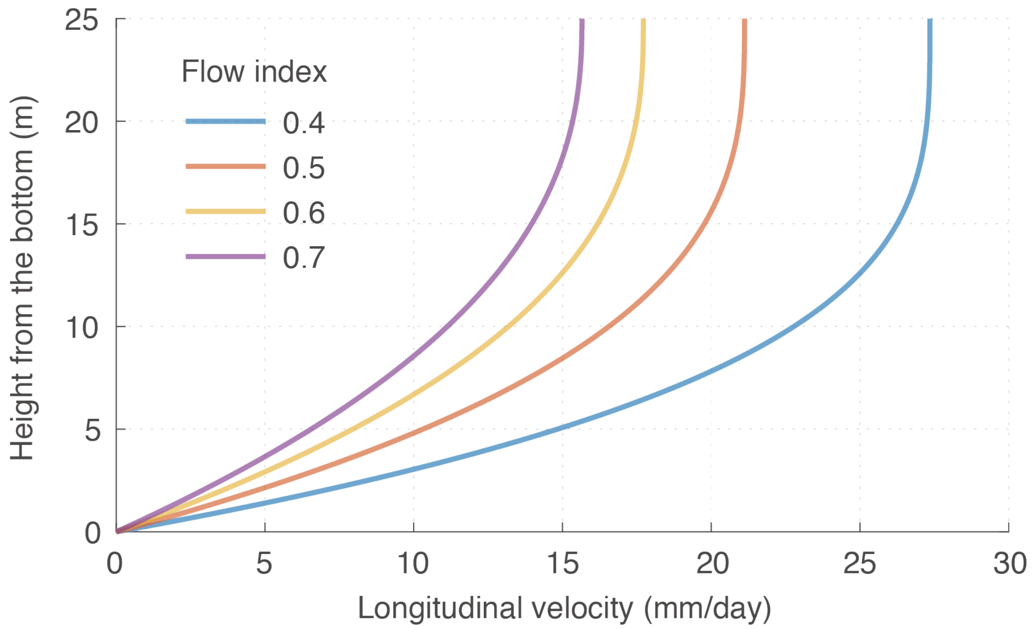

Supplementary Figure 11 | Longitudinal velocity at depth (0-25 m) given the flow index and the best-fit consistency index constrained at the transverse profiles #76 and #77 (Supplementary Table 2).

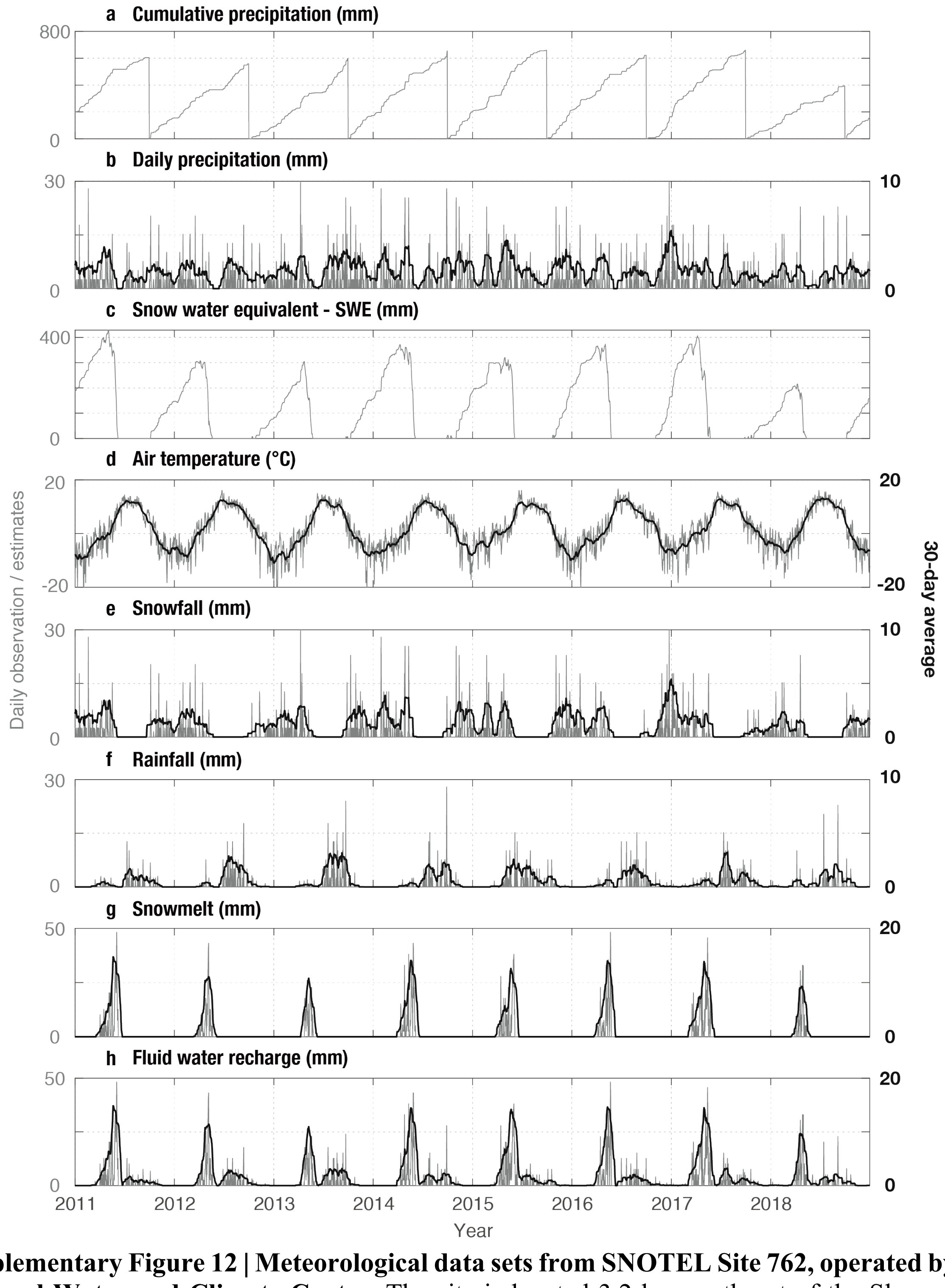

Supplementary Figure 12 | Meteorological data sets from SNOTEL Site 762, operated by U.S. National Water and Climate Center. The site is located 3.2 km southeast of the Slumgullion landslide. **a** to **d** are observations. **e** to **h** are modelled and inferred results. Gray lines represent daily estimates, referring to the left Y axis. Black lines represent 30-day averages using a sliding window, referring to the right Y axis.

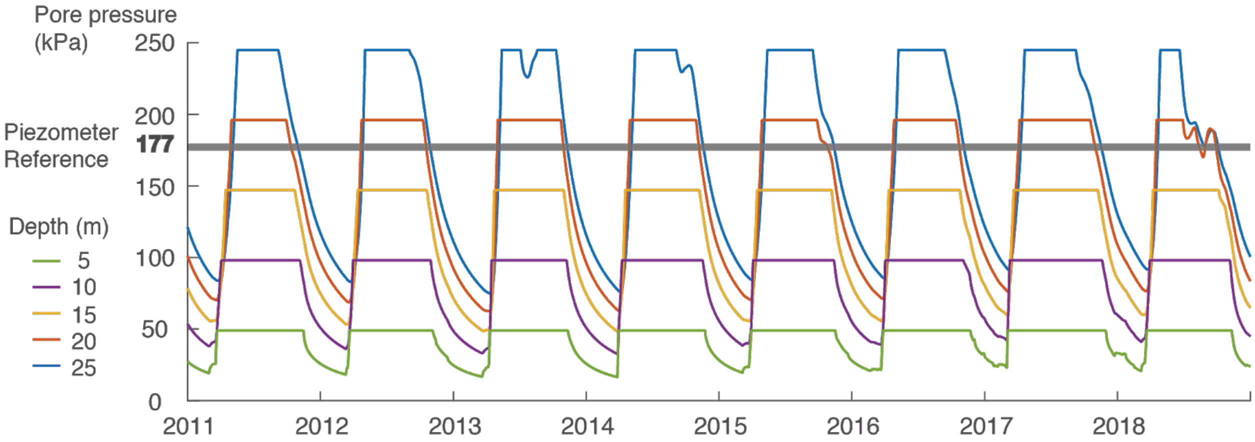

Supplementary Figure 13 | Example of pore pressure time series at different depths given the inferred fluid water recharge (Supplementary Fig. 12h). Horizontal portions of the curves indicate saturation to the ground surface.

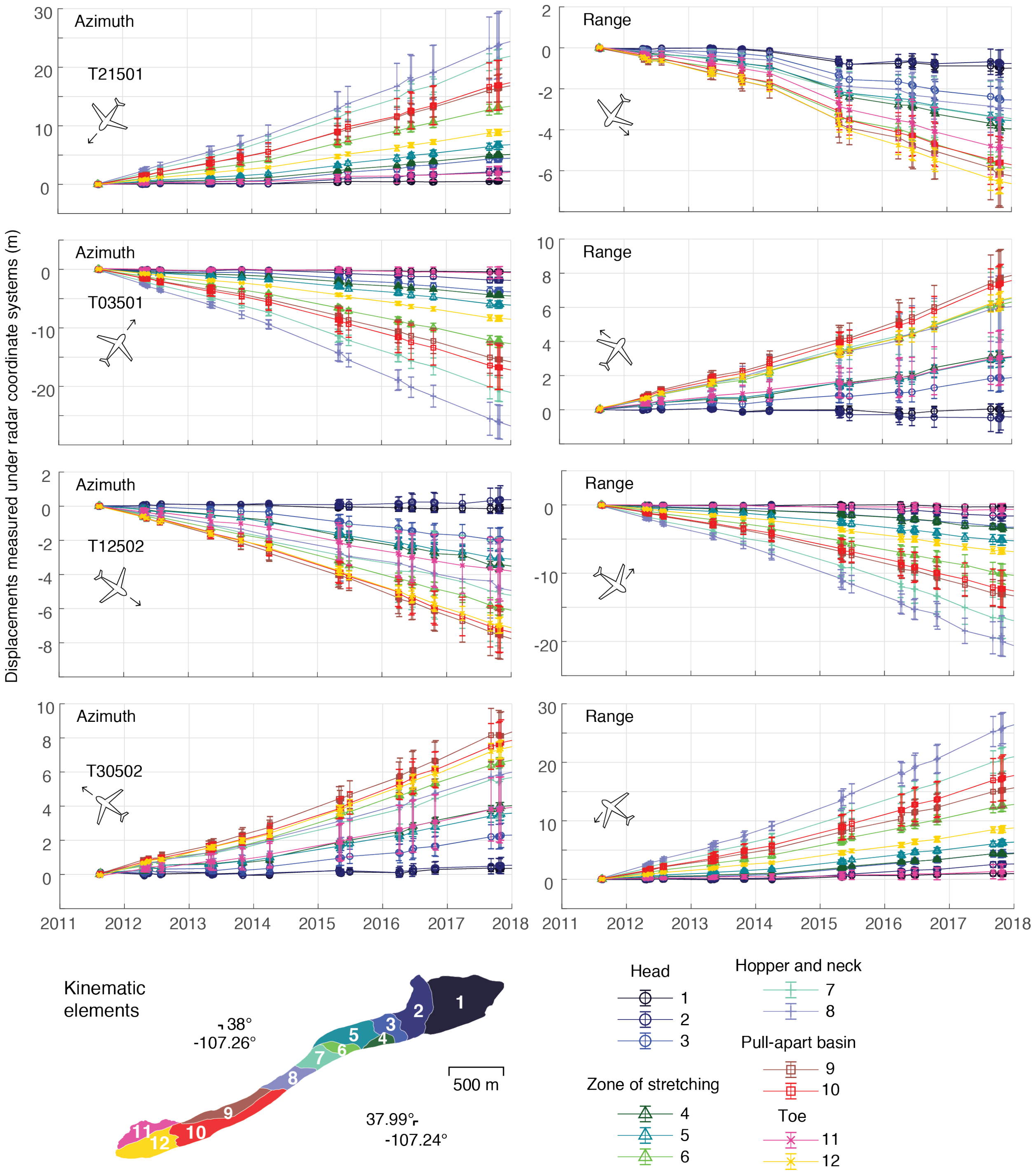

Supplementary Figure 14 | Cumulative landslide displacements for each kinematic element measured along the specified orientations of the UAVSAR measurements.

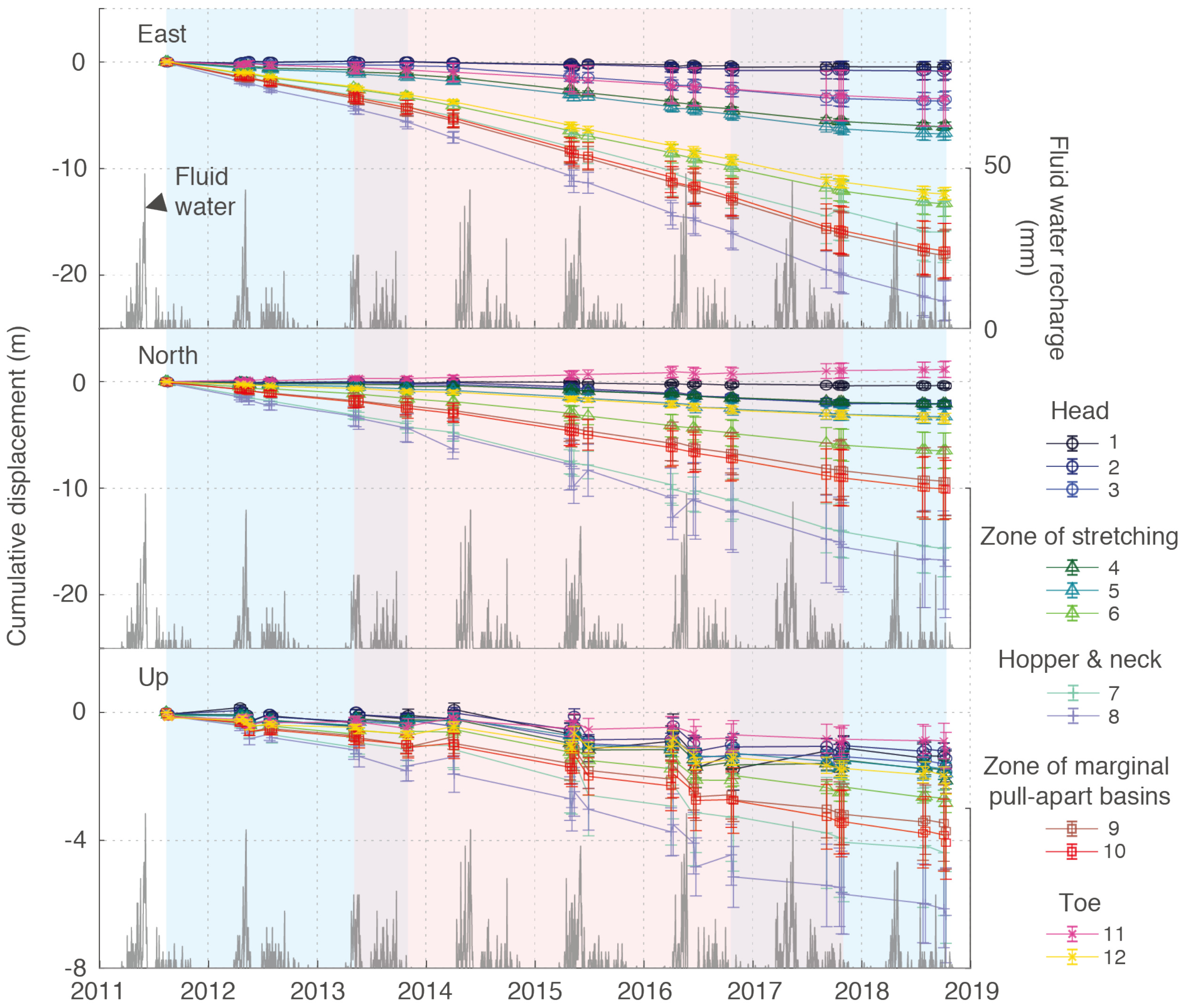

Supplementary Figure 15 | 3D cumulative displacement for each kinematic element in indicated colors from UAVSAR hybrid InSAR-POT method. Blue and red shades on the background show the time periods of deceleration and acceleration, respectively, inferred from the meteorological data and validated by the exponential model. Gray lines show the estimated fluid water recharge at the surface.

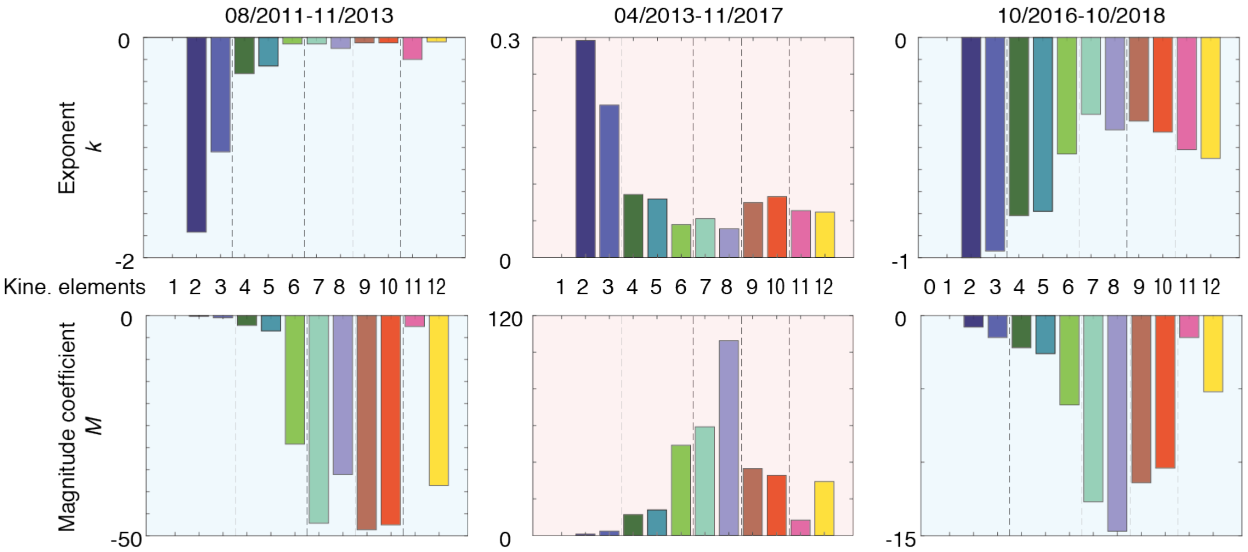

Supplementary Figure 16 | Exponent and magnitude coefficient of the exponential model for kinematic elements in three multi-annual phases.

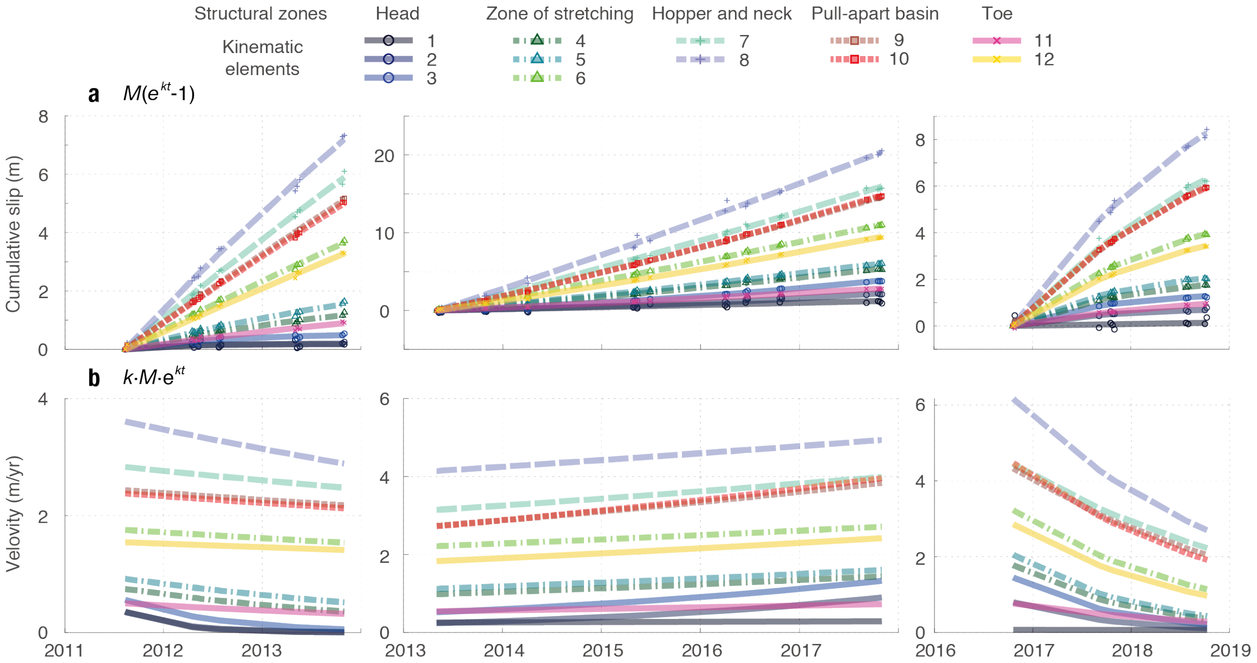

Supplementary Figure 17 | Multi-year slips fitted by an exponential sensitivity model. a, Cumulative slip. Symbols are SAR-derived slip. Thick lines are exponential model fits. **b**, Velocity. Colors represent the different kinematic elements.

Supplementary Table 1. SAR data sets and image pairs.

<table>
<tr><th rowspan="2">Sensor</th><th rowspan="2">Orbit direction</th><th rowspan="2">Track</th><th rowspan="2">Method</th><th colspan="2">Head</th><th colspan="2">Toe</th></tr>
<tr><th># of images</th><th># of pairs</th><th># of images</th><th># of pairs</th></tr>
<tr><td rowspan="2">Sentinel-1</td><td>Ascending</td><td>T49</td><td rowspan="2">InSAR</td><td>45</td><td>70</td><td>28</td><td>32</td></tr>
<tr><td>Descending</td><td>T56</td><td>53</td><td>80</td><td>29</td><td>29</td></tr>
<tr><th rowspan="2"></th><th rowspan="2"></th><th rowspan="2"></th><th rowspan="2"></th><th colspan="4">Entire active landslide</th></tr>
<tr><th colspan="2"># of images</th><th colspan="2"># of pairs</th></tr>
<tr><td rowspan="8">UAVSAR</td><td rowspan="2">Fly along slide</td><td>T21501</td><td rowspan="4">InSAR</td><td colspan="2">24</td><td colspan="2">14</td></tr>
<tr><td>T03501</td><td colspan="2">23</td><td colspan="2">14</td></tr>
<tr><td rowspan="2">Fly across slide</td><td>T12502</td><td colspan="2">25</td><td colspan="2">14</td></tr>
<tr><td>T30502</td><td colspan="2">30</td><td colspan="2">18</td></tr>
<tr><td rowspan="2">Fly along slide</td><td>T21501</td><td rowspan="4">Pixel offset tracking (POT)</td><td colspan="2">29</td><td colspan="2">161</td></tr>
<tr><td>T03501</td><td colspan="2">30</td><td colspan="2">176</td></tr>
<tr><td rowspan="2">Fly across slide</td><td>T12502</td><td colspan="2">31</td><td colspan="2">197</td></tr>
<tr><td>T30502</td><td colspan="2">34</td><td colspan="2">233</td></tr>
</table>

Supplementary Table 2. Best-fit consistency index for given flow index of the power-law flow model.

Flow index n	Consistency index K (Pa·s^n)			Residual (mm/day)	
	#76	#77	Mean	#76	#77
0.4	5.24E+07	3.51E+07	4.37E+07	0.3296953	0.136039
0.5	3.54E+08	2.31E+08	2.92E+08	0.329656	0.1347712
0.6	2.41E+09	1.54E+09	1.97E+09	0.3297495	0.1341436
0.7	1.65E+10	1.03E+10	1.34E+10	0.3299619	0.1340483
0.8	9.99E+10	6.90E+10	8.45E+10	0.5130493	0.134381
0.9	9.93E+10	1.00E+11	9.96E+10	22.6753305	10.7633081

Supplementary Table 3. Best-fit geometric parameters of the slope channel for each transverse profile and the corresponding residuals in the longitudinal velocity.

#	Depth (m)	Tilt (°)	Steepness	Residuals (mm/day)	#	Depth (m)	Tilt (°)	Steepness	Residuals (mm/day)
20	8.85	9.74	5.30E-02	9.13E-03	50	21.12	1.52	5.80E-02	3.55E-02
21	9.70	8.76	4.10E-02	9.58E-03	51	21.06	0.98	7.10E-02	2.56E-02
22	10.61	6.33	5.00E-02	1.45E-02	52	21.05	3.47	4.20E-02	3.69E-02
23	11.46	2.00	3.80E-02	1.32E-02	53	21.02	0.30	5.20E-02	3.32E-02
24	12.10	0.27	3.50E-02	1.22E-02	54	20.44	2.43	4.70E-02	4.22E-02
25	12.39	0.80	3.80E-02	1.66E-02	55	20.04	-0.59	5.80E-02	2.86E-02
26	12.23	-3.04	3.60E-02	1.14E-02	56	19.92	3.26	4.10E-02	2.74E-02
27	12.46	0.03	4.30E-02	1.40E-02	57	20.09	1.10	4.80E-02	3.87E-02
28	12.80	-3.03	3.00E-02	1.86E-02	58	19.67	2.37	6.20E-02	3.14E-02
29	13.08	-0.13	1.60E-02	2.45E-02	59	19.77	0.74	4.70E-02	3.64E-02
30	13.35	5.52	3.60E-02	2.18E-02	60	19.51	3.84	5.80E-02	2.43E-02
31	14.31	9.74	2.80E-02	2.15E-02	61	19.45	3.82	5.10E-02	2.92E-02
32	14.73	14.94	2.90E-02	2.84E-02	62	19.30	5.04	5.70E-02	3.29E-02
33	15.44	15.60	3.80E-02	3.03E-02	63	18.77	1.34	6.60E-02	3.13E-02
34	15.79	17.78	4.30E-02	3.54E-02	64	18.27	0.92	5.40E-02	2.56E-02
35	17.74	11.44	3.40E-02	4.22E-02	65	17.67	0.48	5.90E-02	1.43E-02
36	20.45	3.37	3.00E-02	4.24E-02	66	17.27	0.37	5.30E-02	2.00E-02
37	20.66	4.62	5.70E-02	3.25E-02	67	16.98	-0.90	4.90E-02	2.24E-02
38	21.34	3.62	4.10E-02	3.51E-02	68	16.32	-0.29	4.10E-02	1.58E-02
39	22.04	1.39	4.20E-02	6.52E-02	69	16.22	0.33	4.30E-02	2.58E-02
40	23.00	2.65	4.10E-02	4.27E-02	70	16.35	-0.23	3.10E-02	1.73E-02
41	23.66	0.81	2.50E-02	4.27E-02	71	16.14	1.15	2.80E-02	2.87E-02
42	23.35	0.52	2.90E-02	2.09E-02	72	15.86	7.19	1.90E-02	3.83E-02
43	22.80	0.58	4.40E-02	2.24E-02	73	15.50	8.21	1.40E-02	4.22E-02
44	22.60	4.91	3.70E-02	4.01E-02	74	15.17	9.77	1.60E-02	4.23E-02
45	22.48	1.63	4.70E-02	4.73E-02	75	14.98	11.06	2.00E-02	3.24E-02
46	21.81	1.05	4.70E-02	4.71E-02	76	17.37	4.82	2.50E-02	3.30E-02
47	21.16	0.89	5.40E-02	4.12E-02	77	12.93	7.18	2.90E-02	1.34E-02
48	22.15	-0.62	4.90E-02	4.71E-02					
49	21.40	1.22	4.50E-02	4.68E-02					
