# Supplementary material for: Four-dimensional surface motions of the Slumgullion landslide and quantification of hydrometeorological forcing
Source: Nat Commun. 2020 Jun 3;11:2792. doi: 10.1038/s41467-020-16617-7 (PMC7270131; doi:10.1038/s41467-020-16617-7)
Supplement: Supplementary file 3 — Description of Additional Supplementary Files [file 41467_2020_16617_MOESM3_ESM.pdf]

Description of Additional Supplementary Files

File Name: Supplementary Data 1
Description: Inclinometer data. The 16-element array of 305 mm long tilt sensors (3×10^{-3} mm displacement resolution, long-term accuracy of 0.23 mm) were installed within a polyvinyl chloride-cased borehole. Displacements were measured between September 4, 2016 and October 17, 2016 at 4.93-9.81 m below the ground surface, and between October 19, 2016 and December 7, 2016 at 0-4.88 m below the ground surface (Supplementary Fig. 6). Two data arrays are arranged in time, depth and displacements in millimeters for 16 sensors.

File Name: Supplementary Data 2
Description: Extensometer data. Three extensometers are deployed in different parts of the slide (Fig. 1a). Downhill slips were measured using high-tension linear extension transducers with 0.7 mm accuracy and 8×10^{-4} mm resolution. These instruments are anchored on and off the landslide across its left lateral bounding fault and aligned such that extension occurs parallel to the fault and the landslide's movement, so movement is measured directly. Data are described by dates and displacements in millimeters for three stations.

File Name: Supplementary Movie 1
Description: Surface and subsurface longitudinal velocity, channel geometry, along the transverse cross-sections from the head to the toe of the Slumgullion landslide.
